# Development of Alzheimer’s disease risk score for future integrated primary care: a white-box approach

**DOI:** 10.3389/fnagi.2026.1759273

**Published:** 2026-04-10

**Authors:** Yumiko Wiranto, Devin Setiawan, Amber Watts, Arian Ashourvan

**Affiliations:** 1 Department of Psychology, University of Kansas, Lawrence, KS, United States; 2 Department of Electrical Engineering and Computer Science, University of Kansas, Lawrence, KS, United States; 3 Alzheimer’s Disease Research Center, University of Kansas, Fairway, KS, United States

**Keywords:** Alzheimer’s disease, cognition, integrated health care, interpretable machine learning, risk scores

## Abstract

**Introduction:**

Receiving timely Alzheimer’s disease (AD) diagnosis is often delayed due to long waitlists for specialists. Our study aimed to bridge the gap between the timeliness and complexity of diagnosing AD by developing a scoring system with interpretable machine learning using variables that are obtainable at integrated primary care settings.

**Methods:**

We trained the model using 666 participants with normal cognition or mild cognitive impairment at baseline visit from the Alzheimer’s Disease Neuroimaging Initiative (ADNI) and externally validated the scorecard using 4,876 participants from the National Alzheimer’s Coordinating Center (NACC). We integrated cognitive measures, daily functioning measured with Functional Assessment Questionnaire (FAQ), and demographics into FasterRisk algorithm.

**Results:**

Combinations of 4 separate measures were selected to generate 10 scorecards, showing strong performance (area under the curve [AUC] = 0.868–0.892) in ADNI and remaining robust when externally validated in NACC (AUC = 0.795). The features were Category Animal ≤ 20 (2 points), Trail Making Test B ≤ 143 (−3 points), Logical Memory Delayed ≤ 3 (4 points), Logical Memory Delayed ≤ 8 (3 points), and FAQ ≤ 2 (−5 points). The probable AD risk increased correspondingly with higher total points: 7.4% (−8), 25.3% (−4), 50% (−1), 74.7% (2), and >90% (>6). We refer to this model as the (F)unctioning, (LA)nguage, (M)emory, and (E)xecutive functioning or FLAME scorecard.

**Interpretation:**

Our findings highlight the potential to predict AD development using obtainable information, allowing for implementation into integrated primary care workflows to initiate early intervention. While our scope centers on AD, this established foundation paves the way for other types of dementia.

## Introduction

As the prevalence of Alzheimer’s disease (AD) continues to rise, timely early detection becomes increasingly urgent. One potential barrier to timely diagnosis is the initial point of contact for many patients: their primary care physicians (PCPs). When individuals first notice memory-related issues, the first healthcare professional they typically go to is their PCPs. However, many PCPs may not feel confident in delivering a conclusive diagnosis, resulting in delays in obtaining appropriate care (Bradford et al., 2009; Koch et al., 2010). Additionally, even if PCPs refer their patients for further evaluations, the waitlist for specialists is long, and patients may miss a critical window for early interventions or mitigations of risk factors for AD. Therefore, the solution lies in bridging this diagnostic gap at the primary care level by developing an easily administered and interpretable method to screen for AD risk and involving behavioral health consultants (BHCs), typically clinical psychologists, to carry out the screening and collaborate with PCPs to mitigate the risk factors.

The advancement of machine learning models offers a vast avenue for aiding the diagnostic process due to their speed and data-driven decisions that often excel in comparison to humans (Topol et al., 2019). Recent efforts to develop machine learning models to assist clinicians in identifying early-stage AD have demonstrated robust accuracy (Helaly et al., 2022; Kavitha et al., 2022). However, the use of these models has raised important issues pertaining to the needs for inaccessible data and interpretability due to their “black box” nature in generating the output, potentially leading to a lack of trust in the outputs from clinicians (LeCun et al., 2015; Friedman, 2001). Interpretable machine learning models (IML; i.e., white-box approach), on the other hand, do not suffer from the same issues as it provides the “why” of outputs, offering insights into how specific features contribute to predictions and allowing for transparent and understandable decision-making processes, which has been shown to promote trust between clinicians and the machine learning outputs (Nasarian et al., 2024).

In this study, we developed risk scores that were presented in a scorecard model to assess the risk of developing AD using the FasterRisk algorithm. Risk scores are predictive models that have been used in various fields, including medicine, to aid decision-making processes through basic mathematical calculation (Moreno et al., 2005; Six et al., 2008; Than et al., 2014). The FasterRisk algorithm is a recent advancement that significantly improves the creation of high-quality risk scores that exceeds the performance of traditional methods, such as rounding logistic regression coefficients or non-data-driven approaches (Liu et al., 2022). We selected the following variables to develop the scorecards due to their practicability and comprehensive representation of factors influencing AD: demographic information, cognitive tests from various domains, and daily functioning. We designed the scorecards to help inform clinicians of the probable risk of developing AD based on a patient’s presentation, aiding in decisions about when to refer patients to specialists and involving BHCs to initiate interventions while the patients are on the waitlist for specialists. We predicted that our framework could generate a scoring system with robust predictive power using accessible variables.

## Materials and methods

### Participants

We included data from 676 baseline visits from several Alzheimer’s Disease Neuroimaging Initiative database (ADNI 1, 2, GO, and 3; adni.loni.usc.edu) as of August 2023 and 4,876 baseline visits from the National Alzheimer’s Coordinating Center (NACC) Uniform Data Set (UDS) extracted in March 2024. ADNI was launched in 2003 as a public-private partnership, led by Principal Investigator Michael W. Weiner, MD. The primary goal of ADNI has been to test whether serial magnetic resonance imaging (MRI), positron emission tomography (PET), other biological markers, and clinical and neuropsychological assessment can be combined to measure the progression of mild cognitive impairment (MCI) and early AD.

ADNI’s broader criteria include age 55–90, a minimum of 6 years of education, consistent medication for the past 4 weeks, Hachinski scale < 4 (to rule out vascular dementia), and Geriatric Depression Scale < 6; more information can be found www.adni-info.org. We conducted performance validation of the model using an independent cohort, NACC UDS, which is a multi-site study that has been collecting data from Alzheimer’s Disease Research Centers (ADRCs) since 2005. In general, NACC UDS inclusion criteria for enrollment include an absence of neurological disorders, severe mental illness, learning disability, and substance abuse. The data consists of participants’ demographics, cognitive status, cognitive performance, and other clinical history and information; more information can be found www.naccdata.org or prior publications detailing the recruitment process and data collection (Beekly et al., 2004; Beekly et al., 2007).

We included participants in our analysis who were classified by ADNI and NACC as having normal cognition (NC) or amnestic MCI. Participants were divided into two groups: stable and progressive. The stable group consisted of individuals who remained at the same diagnosis level over time. The progressive group included those who developed AD at later visits. Specifically, participants who progressed from aMCI to AD or NC to AD were placed in the progressive group. To be included in the stable group, the sample had to contain data indicating at least 3 years of aMCI and 8 years of NC. Individuals who reverted to aMCI from NC were not included in the analysis. Participants from both NC and MCI baseline groups were pooled into a single validation cohort to reflect the continuous nature of the Alzheimer’s disease spectrum and to ensure the scorecard acts as a robust and stage-agnostic tool for early risk detection. We excluded participants with non-amnestic MCI or those who developed other types of dementia.

Participants with invalid or missing values were identified and removed from ADNI (*n* = 10; 1.48%). The pattern of missingness in the NACC dataset was evaluated using standardized mean differences, and the result suggested that it is not missing completely at random. To address the systematic missingness in the NACC dataset, we employed Multiple Imputation by Chained Equations (MICE), implemented via the IterativeImputer framework. This approach models each feature with missing values as a function of other features in an iterative round-robin fashion. We generated *m* = 5 imputed datasets, utilizing a Bayesian posterior sampling approach (sample_posterior = True) to account for the uncertainty of the missing values. The imputation model incorporated all predictive features (LDEL, LOGIMEM, FAQ, TRAILB, ANIMALS) alongside demographic covariates (Age, Sex, Education, Race) to maximize the accuracy of the estimated values.

### Neuropsychological tests and functioning

We selected a range of neuropsychological tests that tapped into a variety of cognitive domains, such as attention, executive function, memory (short-term and long-term), and language/verbal fluency. The selected tests were the Logical Memory Immediate (LIMM), Logical Memory Delayed (LDEL), Category Animal (CATANIMSC), Trail Making Test A (TMT A), and Trail Making Test B (TMT B). These tests were selected because they were administered across all ADNI and NACC cohorts. Additionally, we included the instrumental activities of daily living measured with the Functional Activities Questionnaire (FAQ). Some adjustments were made for the NACC dataset because some tests were replaced or not administered. For example, the later NACC cohorts (UDS v.3) completed Craft Story 21 instead of Logical Memory (UDS v.2). We converted the test scores to match Logical Memory based on the conversion table provided by NACC crosswalk study (Monsell et al., 2016).

### Data preprocessing and encoding

**Figure 1:**
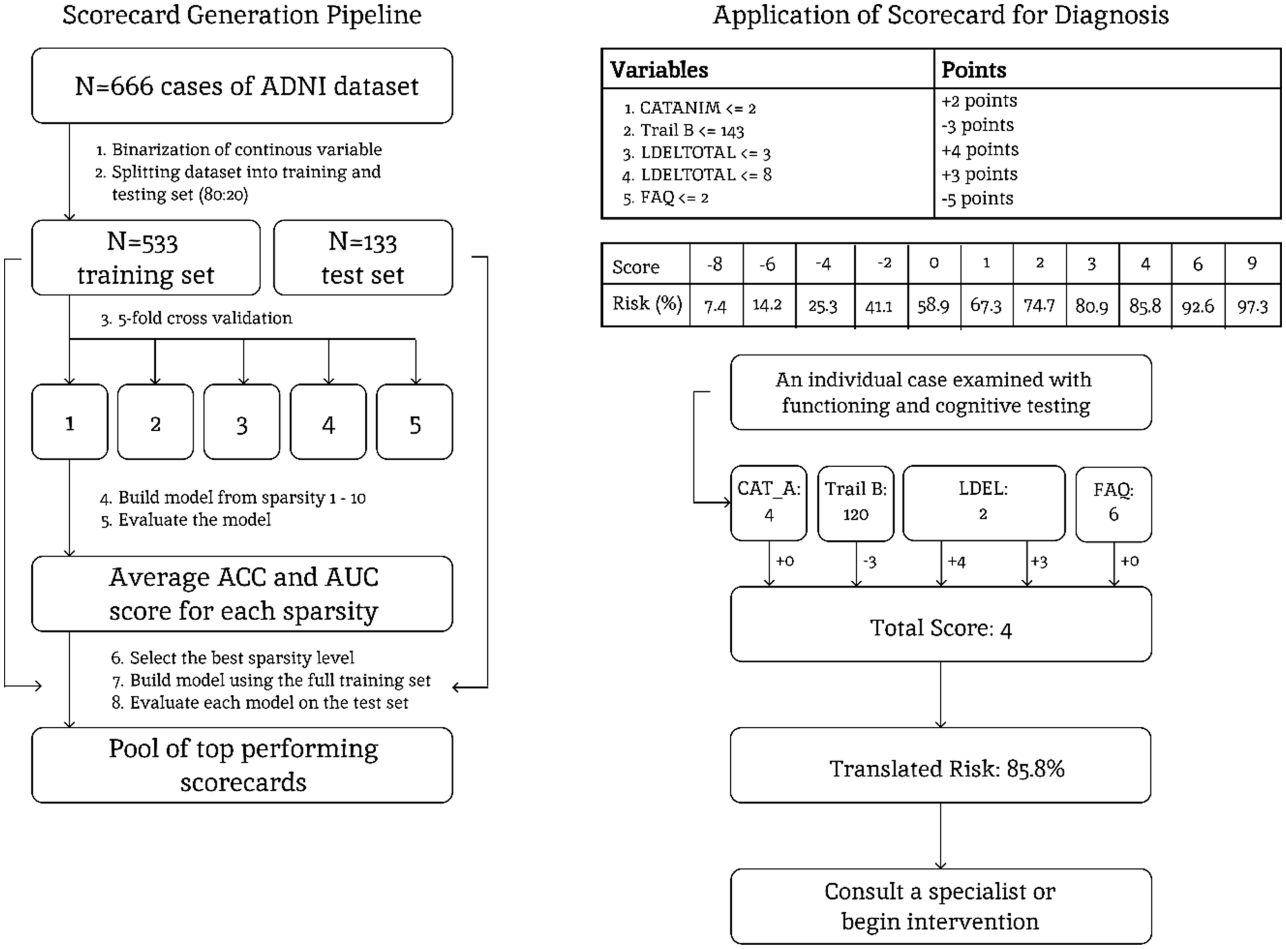
Pipeline of conducting FasterRisk algorithm to generate the FLAME scorecard and its clinical application.

To prepare the data for analysis, we converted categorical variables into numerical representations through Scikit-learn Labelencoder. We opted for label encoding over one-hot encoding because the FasterRisk algorithm is optimized for continuous or ordinal-like inputs that are subsequently binarized. The next preprocessing step was applying binarization using the FasterRisk built-in binarization module to convert the features from continuous into binary features (Figure 1). This process was data-informed rather than clinically derived. The algorithm identifies optimal thresholds by converting each continuous variable into a set of 100 distinct binary features based on the data distribution (e.g., Age ≤ 65, Age ≤ 66, etc.). This fine-grained binarization allows the scorecard to exhaustively explore the space of possible thresholds and select the specific cut-point that maximizes predictive power relative to the outcome variable. This approach is also non-parametric, which ensures that outliers and skewed tails do not disproportionately influence the final risk scores. To prevent data leakage, thresholds and the resulting feature pool were learned strictly on the training data during each fold of the cross-validation process, ensuring that the test data remained entirely unseen during the threshold optimization phase.

### Risk scorecard generation and optimization

The scorecard was trained and internally validated with the ADNI dataset and was externally validated using the NACC dataset. We employed the FasterRisk algorithm to develop an interpretable, point-based risk scorecard, a model type widely used for transparent clinical decision-making (Liu et al., 2022). Specific risk factors are assigned simple, whole-number points (e.g., +5 points for a given condition). A clinician sums up these points to calculate a patient’s total risk score, which corresponds to a risk percentage, making each decision transparent and easy to explain. The algorithm then constructs risk scores through an iterative process. It starts by identifying the single most predictive risk factor to form a base model. Then, it incrementally adds new factors one by one, keeping only those that improve the model’s performance. The algorithm also explores other combinations by swapping existing features with other promising candidates. This approach was selected because it significantly outperforms RiskSlim, a previous state-of-the-art model for finding risk scores (Ustun and Rudin, 2019).

The scorecard’s complexity is controlled by a ‘sparsity’ parameter, k, which dictates the exact number of predictive features included. To find the optimal balance between simplicity and accuracy, we systematically tested models with 1–10 features using stratified 5-fold cross-validation (Figure 1). The optimal sparsity, k, was determined by the highest mean validation Area Under the ROC Curve (AUC) across all five folds. Within this optimal k, the final scorecard was selected from a pool of the top 10 candidate models ranked by their logistic loss on the training data. To ensure the scorecard remained practical, we imposed a sparsity constraint on the number of features and a coefficient constraint (limiting points to the range of [−5, 5]). These constraints act as a form of regularization, preventing overfitting while forcing the model to prioritize only the most robust predictors for the final point-based calculation. While formal feature stability metrics were not the primary selection driver, the iterative search nature of the algorithm naturally prioritized features with the highest selection frequency across the diverse candidate pool.

### Final model evaluation

The final scorecard was trained on 80% of ADNI data and its performance was internally validated on the held-out 20% test set (Figure 1). To ensure the stability of our results, we generated and analyzed the top 10 scorecards produced by the algorithm. By doing this, we were able to examine the set of most consistently predictive features. The model’s performance was evaluated using accuracy, where a 50% risk threshold determined classification, and also the Area Under the Receiver Operating Curve (AUC), which quantifies the model’s overall ability to discriminate between classes.

We further assessed the model’s calibration for estimating absolute progression probability by calculating Brier scores and generating calibration plots. To ensure our findings were not dependent on a single model selection, we validated all 10 candidate scorecards across the imputed NACC sets and report the mean and standard deviation for AUC, Accuracy, and Brier scores across these evaluations. Calibration plots for the remaining scorecards are provided in Supplementary material. Notably, the scorecard’s point-based scoring system and its associated risk-probability mapping were derived solely from the ADNI training set. No retraining, parameter optimization, or scorecard threshold adjustments were performed for the NACC cohort, which served as a strictly independent external validation. All data preprocessing, model building, and evaluation were performed using Python (v3.12.3). The software environment included Pandas (v2.3.3) for data manipulation, NumPy (v2.3.4) for numerical computations, and Scikit-learn (v1.7.2) for label encoding, cross-validation partitioning, and performance metrics. The FasterRisk algorithm was implemented using the official FasterRisk Python package.

## Results

### Participant characteristics

We included data from 666 ADNI participants (164 NC and 502 aMCI) and 4,876 NACC participants (3,540 NC and 1,336 aMCI) from 41 ADRCs at baseline visits. Over time, 43.8% (*n* = 292) of ADNI and 37.4% (*n* = 1823) of NACC participants were diagnosed with AD. As shown in Table 1, ADNI participants were 44.1% female, with a mean age of 73.44 years and an average of 16.15 years of education, and NACC participants were 65.80% female, with a mean age of 73.44 years and an average of 16.15 years of education. There were statistically significant group differences between stable and progressive in ADNI (age at *p* < 0.01 and education at *p* < 0.05) and NACC (age and education at *p* < 0.001). The average transitions for the aMCI-AD and the NC-AD groups are 1.09 years and 7.10 years for ADNI and 3.02 years and 7.20 years for NACC.

**Table 1:** Table of ADNI and NACC (exclude missing data) demographic, cognition, and functioning by groups.

Variables (ADNI)	Stable (*n* = 374)	Progressive (*n* = 292)	All (*n* = 666)
Age (years)	72.8 (6.74) **	74.27 (7.03) **	73.44 (6.90)
Education (years)	16.34 (2.72) *	15.89 (2.82) *	16.15 (2.77)
Female (%)	169 (45.2%)	124 (42.6%)	293 (44.1%)
Race (White%)	350 (93.6%)	274 (94.2%)	624 (93.83%)
FAQ	0.98 (2.56) ***	4.90 (5.00) ***	2.70 (4.29)
Category animal	19.91 (5.43) ***	16.11 (4.94) ***	18.25 (5.55)
TMT A	35.29 (14.42) ***	45.9 (24.2) ***	39.93 (20.01)
TMT B	87.91 (46.57) ***	134.63 (73.83) ***	108.36 (64.32)
LM immediate	11.66 (3.84) ***	7.63 (3.88) ***	9.90 (4.35)
LM delayed	9.83 (4.21) ***	4.59 (4.13) ***	7.54 (4.92)

Variables (NACC)	Stable (*n* = 2,445)	Progressive (*n* = 1,212)	All (*n* = 3,657)
Age (years)	68.16 (9.55)***	76.61 (8.49)***	70.96 (10.03)
Education (years)	16.61 (6.15)***	15.85 (5.6)***	16.36 (5.98)
Female (%)	1,646 (67.3%)	759 (62.6%)	2,405 (65.8%)
Race (White%)	2074 (84.8%)	1,075 (88.7%)	3,149 (86.11%)
FAQ	0.26 (1.33)***	2.59 (4.66)***	1.03 (3.1)
Category animal	21.87 (5.66)***	17.70 (5.66)***	20.49 (5.99)
TMT B	80.35 (79.88)***	140.73 (168.42)***	100.36 (120.29)
LM delayed	12.91 (4.19)***	7.68 (5.26)***	11.18 (5.19)

ADNI, Alzheimer’s Disease Neuroimaging Initiative; NACC, National Alzheimer’s Coordinating Center; FAQ, Functional Activities Questionnaire; TMT A, Trail Making Test A; TMT B, Trail Making Test B; LM, Logical Memory.

Differences between stable and progressive, *p < 0.05, **p < 0.01, ***p < 0.001.

Cognitive measures were adjusted for age and education.

FAQ was adjusted for age.

### Group differences in functioning and cognitive performance at baseline

We compared group differences in cognitive performance and daily functioning using analysis of covariance (ANCOVA) between stable and progressive groups. The FAQ was adjusted for age, and cognitive measures were adjusted for age and education. All measures showed statistical differences between stable and progressive groups in both ADNI and NACC (*p* < 0.001). The progressive groups showed lower performance and scores in verbal fluency/language, TMT A, TMT B, immediate and delayed memory, and daily functioning. Higher values on TMT A and TMT B indicate worse performance.

### Alzheimer prediction risk score

Based on the FasterRisk algorithm, a sparsity level of 5 was selected for the most optimal combination for the generation of the final scorecards to predict AD development. Ten scorecards were generated with a test AUC ranging from 0.868 to 0.892 and accuracy between 81.20 and 84.21% (Supplementary Figure 1). The scorecard with the highest test AUC (0.892) shown in Table 2 represents Category Animal ≤ 20 (2 points), Trail Making Test B ≤ 143 (−3 points), Logical Memory Delayed ≤ 3 (4 points), Logical Memory Delayed ≤ 8 (3 points), and FAQ ≤ 2 (−5 points). Positive points contribute to increased total score of the scorecard, while negative points decrease it. The total score corresponds to the risk percentage shown in Table 2. The probable AD development risk was 7.4% for a total score of −8, 19.1% for a score of −5, 32.7% for a score of −3, 50% for a score of −1, 74.7% for a score of 2, 85.8% for a score of 4, and greater than 90% for a score of 6, 7, or 9 (Table 2). We refer to this scorecard model as the FLAME scorecard, a mnemonic representing functioning, language, memory, and executive function.

**Table 2:** Scorecard with the highest AUC and risk score to assess AD development probability.

Variables	Points	Assigned points
1. Category animal ≤ 20	+ 2 points	…
2. Trail making test B ≤ 143	−3 points	+ …
3. Logical memory delayed ≤ 3	+ 4 points	+ …
4. Logical memory delayed ≤ 8	+ 3 points	+ …
5. FAQ ≤ 2	−5 points	+
	SCORE	=
LDEL = Logical Memory delayed recall; Test; FAQ = Functional Activities Questionnaire.		

Score	−8	−6	−4	−3	−1	1	2	4	6	9
Risk (%)	7.4	14.2	25.3	32.7	50.0	67.3	74.7	85.8	92.6	97.3

**Figure 2:**
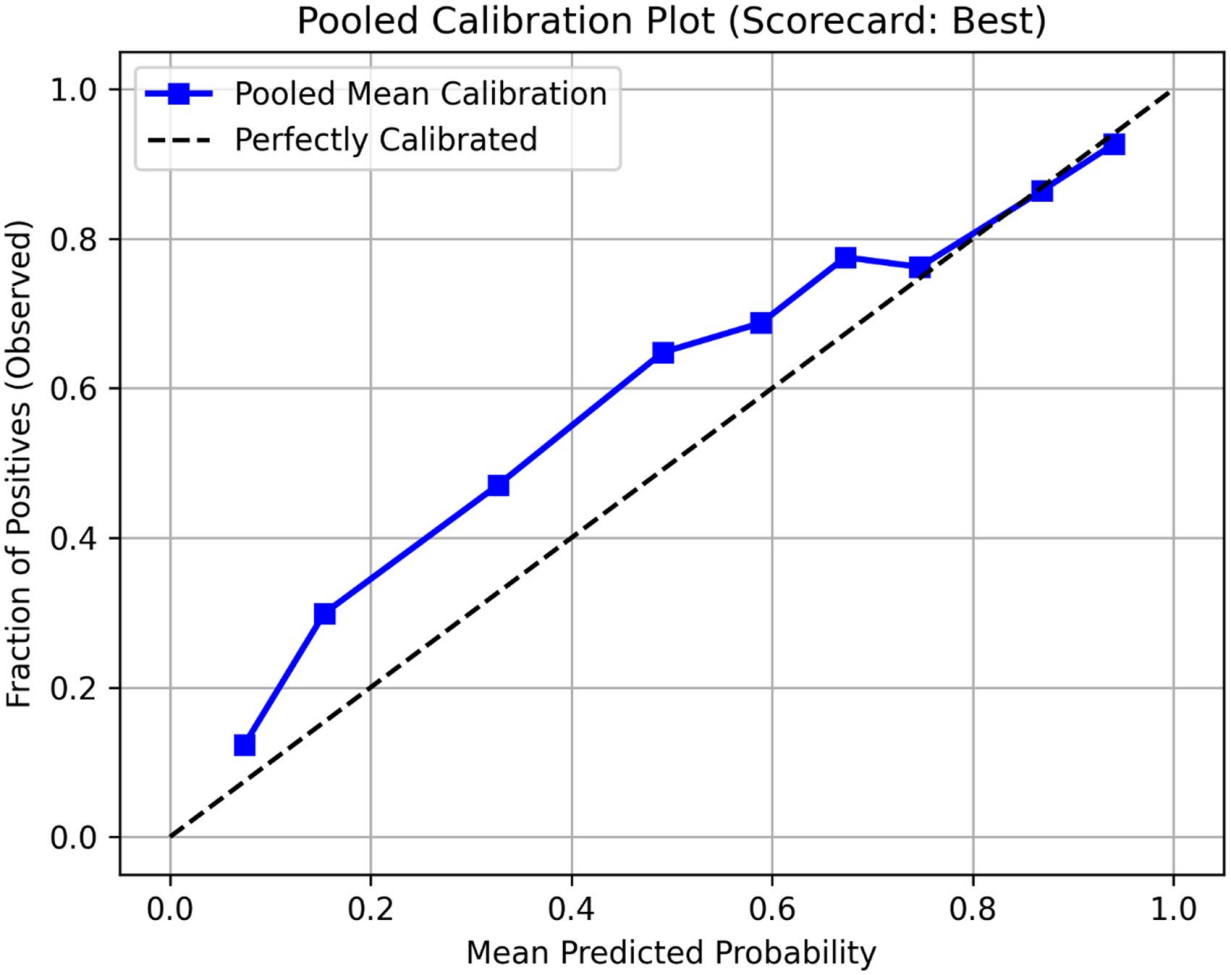
Calibration plot of best scorecard on external NACC dataset.

To ensure the robustness of the results against potential selection bias, the scorecard was validated on the full NACC cohort using MICE across five imputed datasets. The FLAME scorecard demonstrated strong generalizability with a pooled mean AUC of 0.802 (±0.0006) and a Brier score of 0.177 (Table 3). Calibration analysis of the pooled results (Figure 2) showed an excellent agreement between predicted and observed risk, confirming the model’s reliability across the clinical spectrum. Furthermore, stratified analyses were conducted to evaluate performance across baseline clinical stages (Table 4). The results demonstrate that the scorecard maintains high predictive validity in both subgroups. Notably, the AUC for the Normal-only baseline cohort (0.7101 ± 0.0006) was comparable to, and slightly higher than, the MCI-only cohort (0.7052 ± 0.0008). This indicates that the scorecard is not merely picking up on advanced impairment in MCI subjects but is successfully identifying early markers of progression even in cognitively normal individuals. Furthermore, the high accuracy (78.7%) and superior Brier score (0.1626) in the Normal group underscore the model’s utility as an early screening tool. These findings suggest that pooling the groups does not obscure risk dynamics but rather highlights a shared predictive signature across the Alzheimer’s clinical continuum.

**Table 3:** Table of mean and standard deviation of performance metrics on five imputed NACC dataset.

Performance metric	Best	#1	#2	#3	#4	#5	#6	#7	#8	#9
AUC	0.802 ± 0.0006	0.801 ± 0.0005	0.802 ± 0.0005	0.801 ± 0.0005	0.801 ± 0.0005	0.800 ± 0.0006	0.800 ± 0.0006	0.800 ± 0.0005	0.801 ± 0.0006	0.796 ± 0.0006
ACC	0.763 ± 0.0007	0.763 ± 0.0007	0.764 ± 0.0007	0.763 ± 0.0007	0.764 ± 0.0007	0.763 ± 0.0007	0.763 ± 0.0007	0.762 ± 0.0007	0.755 ± 0.0005	0.757 ± 0.0006
Brier	0.177 ± 0.0003	0.176 ± 0.0003	0.175 ± 0.0003	0.176 ± 0.0003	0.175 ± 0.0003	0.177 ± 0.0003	0.177 ± 0.0003	0.176 ± 0.0003	0.175 ± 0.0003	0.180 ± 0.0003

**Table 4:** Table of performance comparisons between different baseline group.

Stratum	AUC	ACC	Brier score
Full cohort	0.802 ± 0.0006	0.763 ± 0.0007	0.177 ± 0.0003
Normal only	0.710 ± 0.0006	0.787 ± 0.0006	0.163 ± 0.0003
MCI only	0.705 ± 0.0008	0.699 ± 0.0009	0.214 ± 0.0004

## Discussion

Our study presents a novel approach to predicting the risk of developing AD that offers potential to be applied in integrated primary care settings by employing a set of obtainable variables, including demographics, daily functioning, and cognitive performance. By utilizing the FasterRisk algorithm, we generated 10 scorecards, each achieved high predictive accuracy (81.20–84.21%) and strong discriminative performance (AUC = 0.868–0.892), reflecting a robust balance between sensitivity and specificity in identifying individuals at risk of developing AD. We further validated one of the 10 scorecards on a separate cohort, indicating good generalizability. Additionally, the FLAME scorecard compared favorably to established machine learning models for predicting AD development (Fraser et al., 2016; Ye et al., 2012) and compared similarly or slightly below previously studied interpretable machine learning models incorporating biological (AUC = 0.86) and neuroimaging data (accuracy = 87.5%) (Vlontzou et al., 2025; Das et al., 2019).

A key advantage of the FLAME scorecard lies in its potential clinical practicality at integrated primary care settings. We selected variables that can be implemented and fit in one appointment with a behavior health consultant (BHC), which typically lasts approximately 30 min. The BHC would also have sufficient time to gather brief information related to patients’ concerns and general mental wellbeing. Following this assessment, the BHCs can input the results into the scorecard and collaborate with the PCPs to determine appropriate next steps, which may vary depending on the findings and presenting concerns. For instance, the PCPs may refer patients to neurologists or other specialists, start a pharmacological treatment to slow down memory decline (e.g., acetylcholinesterase inhibitors), reduce the use of anticholinergic medications, order p-tau 217 blood test, or recommend patients to make follow-up appointments with the BHCs to address behavioral factors that could mitigate memory decline (e.g., sleep hygiene, engaging in physical activity, medication adherence, mood symptoms). BHCs may also address safety concerns (e.g., driving, living arrangement) and make appropriate recommendations. These services could serve as valuable interim interventions while patients are placed on the waitlist for specialists.

Another advantage of the FLAME scorecard lies in its interpretability, which provides clinicians with a clear explanation of the specific features influencing its predictions. This transparency promotes human-computer interaction, in this case, trust between clinicians and the machine learning outputs (Nasarian et al., 2024). Furthermore, these scorecards offer flexibility in their implementation, which allows clinicians to incorporate their expertise into the scorecards when predicting the risk of AD development. For example, on the FLAME scorecard, having a FAQ score of 2 or below would decrease the total by 5 points. If a patient indicates that the reason for requiring assistance on some activities is due to physical limitations rather than decreased mental capacity, then the clinician can consider this context when evaluating the predicted risk of AD. The balance between performance, interpretability, and flexibility positions our scorecard as a promising tool for practical clinical application, where understanding the rationale behind predictions is paramount for effective and informed decision support.

There are some limitations in our study. The scorecards generated in this study are only applicable to typical AD with an amnestic profile. Recent reviews highlighted that a subset of AD populations presents an atypical profile, such as deficits in executive functioning, language, visuospatial, and other non-memory domains, and this subset is typically younger than those who develop typical AD (Graff-Radford et al., 2021; Shea et al., 2021). Another limitation is that we did not include neuropsychiatric symptoms (NPS) in model development. Research has shown that symptoms, such as depression, anxiety, apathy, and agitation, may emerge in the early stages of MCI and increase the incidence of progression to AD or dementia (Rosenberg et al., 2013; Hu et al., 2020). Excluding these symptoms may limit generalizability and model performance in predicting conversion to AD, particularly for individuals who exhibit psychiatric symptoms prior to cognitive decline. This scorecard might also be limited to discern typical AD presentation from limbic predominant age-related TDP-43 encephalopathy (LATE) due to similarity in their cognitive profiles (Kapasi et al., 2020; Serio et al., 2025). Future studies including participants with atypical profiles or LATE are necessary to better inform PCPs of which follow-up tests to order to help confirm their diagnosis, which will cut down some costs compared to sending the patients to all tests/procedures.

The demographic composition of the ADNI and NACC samples consists of predominantly White and highly educated individuals. Research has shown that Black individuals and those with lower educational levels demonstrate lower cognitive performance at baseline, but the former group show a slower decline compared to their White counterparts (Weuve et al., 2018; Wilson et al., 2015). Additionally, those who were born outside of the United States may be at disadvantage in terms of their performance on the tests that were designed to measure cognitive functioning in Western populations (Meunier et al., 2025; Liu et al., 2025). Given that our scorecard heavily relies on cognitive performance, it may demonstrate reduced sensitivity and specificity in the aforementioned populations. External validation and recalibration in these populations will be imperative before clinical implementation. Lastly, it was beyond the scope of our study to determine a definitive cutoff value for clinical decision-making.

Our study lays the groundwork for a more accessible and population-wide approach to screening for Alzheimer’s disease. Moving forward, we aim to collaborate with primary care physicians and behavioral health consultants to collect both qualitative and quantitative data on the feasibility and potential impact of implementing these scorecards in routine clinical practice. This collaboration will provide valuable insights into the practical challenges and opportunities for integrating our tool into the healthcare system.

## Conclusion

Our study generated a robust scoring system for predicting the likelihood of developing Alzheimer’s disease using accessible and cost-efficient variables through interpretable machine learning. This framework’s interpretability may aid clinicians in integrated primary care settings in providing early detection to their patients, including those residing in resource-constrained areas.

## Data Availability

Data access can be requested on the ADNI (adni.loni.usc.edu) and NACC (www.naccdata.org) website.

## Group member of the Alzheimer’s Disease Neuroimaging Initiative

Data used in preparation of this article were obtained from the Alzheimer’s Disease Neuroimaging Initiative (ADNI) database (adni.loni.usc.edu). As such, the investigators within the ADNI contributed to the design and implementation of ADNI and/or provided data but did not participate in analysis or writing of this report. A complete listing of ADNI investigators can be found at: http://adni.loni.usc.edu/wp-content/uploads/howtoapply/ADNI_Acknowledgement_List.pdf.
